# A case of laparoscopic partial hepatic S7 resection for postoperative liver metastasis of rectal malignant melanoma

**DOI:** 10.1186/s40792-021-01316-2

**Published:** 2021-10-26

**Authors:** Makoto Takahashi, Yasuhiro Morita, Tatsuya Hayashi, Susumu Yanagibashi, Shunsuke Sato, Shu Sasaki, Kunio Takuma, Haruka Okada

**Affiliations:** 1 Department of Surgery, Tokyo Metropolitan Tama Medical Center, 2-8-29 Musashidai, Fuchu, Tokyo 183-8524 Japan; 2 Department of Pathology, Tokyo Metropolitan Tama Medical Center, Tokyo, Japan

**Keywords:** Anorectal malignant melanoma, Liver metastasis, Laparoscopic surgery

## Abstract

**Background:**

Anorectal malignant melanoma (ARMM) has an extremely poor prognosis, and there is no report of resection of liver metastases so far. We report herein a rare case of postoperative laparoscopic partial hepatic S7 resection for rectal malignant melanoma.

**Case presentation:**

A 51-year-old female patient with a diagnosis of an ARMM underwent a laparoscopic rectal amputation. Eleven months later, computed tomography (CT) revealed a 14-mm nodule in liver segment 7 (S7), which was diagnosed as a hepatic recurrence of the ARMM. Because no other recurrences were found, a laparoscopic partial resection of S7 was performed. Pathological analysis found intracellular melanin deposition, and immunostaining was S-100 (+), HMB-45 (+), and SOX-10 (+). Based on these findings, a liver metastasis of malignant melanoma was diagnosed. The patient is alive 7 months after the second surgery and has so far experienced no recurrences.

**Conclusion:**

We reported an extremely rare case of a laparoscopic resection of a liver metastasis following surgery for ARMM.

## Background

Anorectal malignant melanoma (ARMM) is a rare disease, accounting for about 1% of all malignant melanoma cases and 0.5–2% of all anorectal malignancies [1]. It is highly malignant, with a 5-year survival rate of 25% and a median survival of 16 to 28 months [2–4]. Even if radical resection is performed for ARMM, recurrences in the liver and lung are highly likely. Herein, we described a rare case of a laparoscopic resection of a liver metastasis following surgery for ARMM.

## Case presentation

**Fig. 1:**
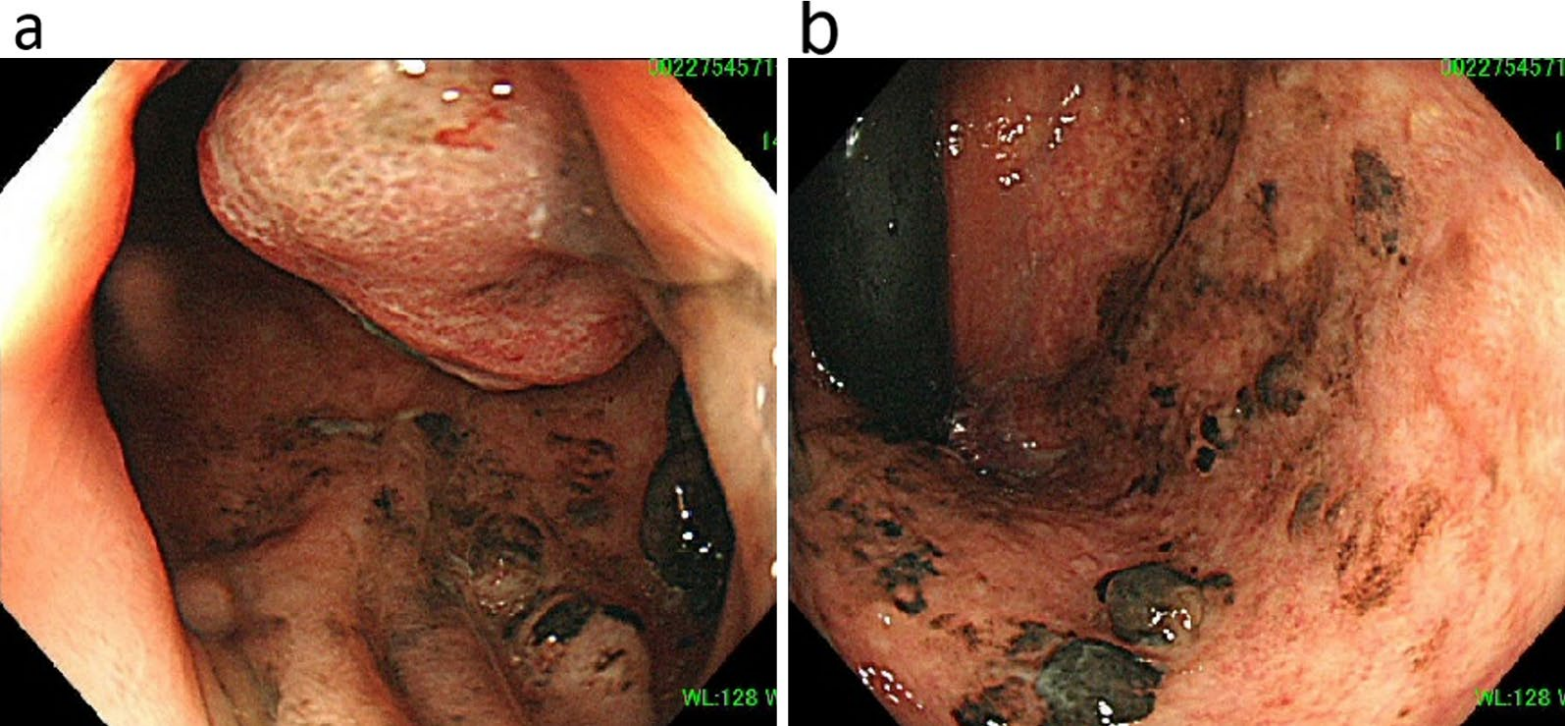
Colonoscopy. **a** Sub-pedunculated tumor found in the lower rectum (Rb). **b** Black protruding lesion found extending upwards into the anal canal

**Fig. 2:**
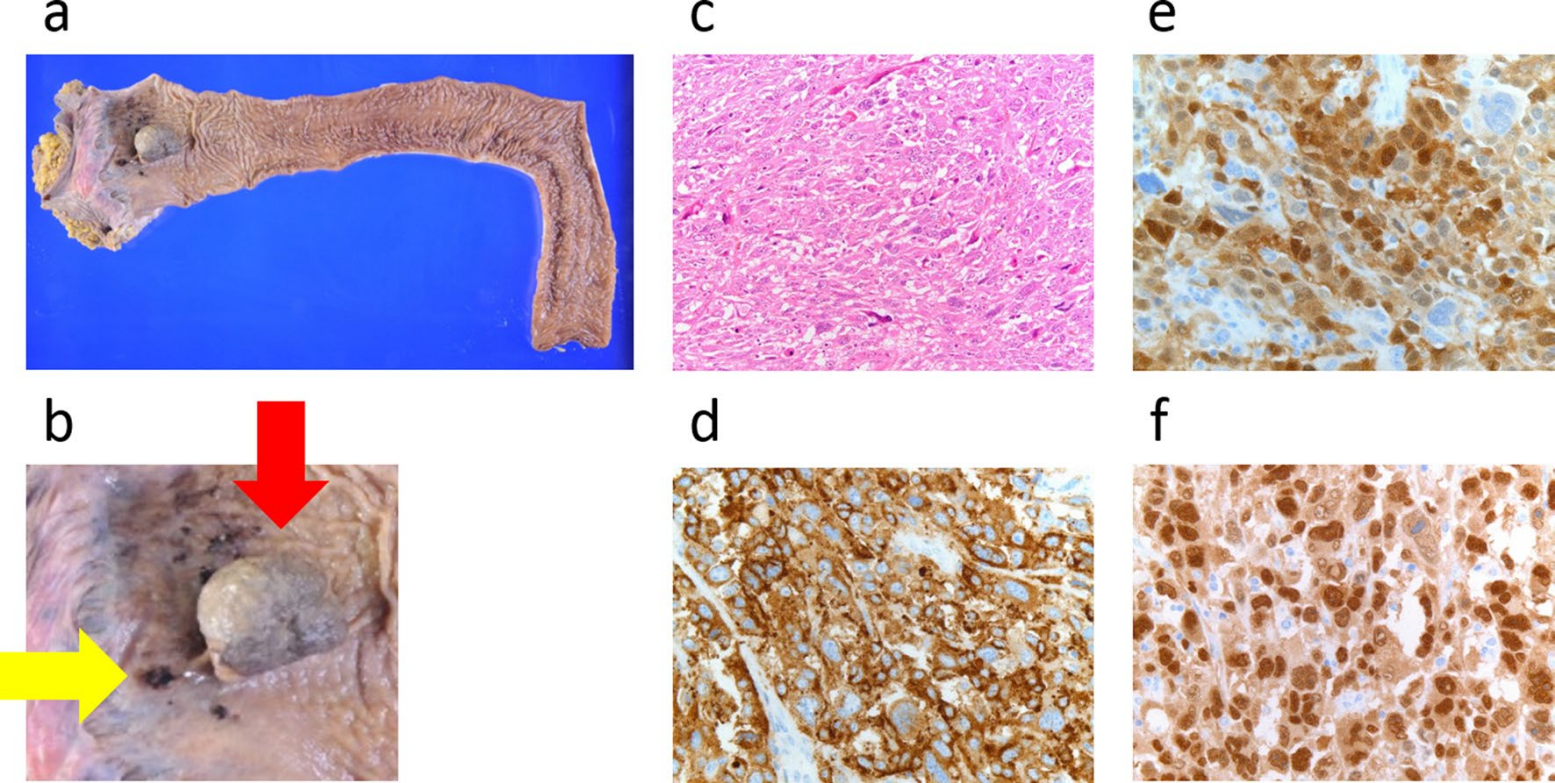
Macroscopic findings, histopathological findings, and immunostaining findings of ARMM. **a**,** b** Pathological specimens revealed a semi-pedunculated tumor 30 mm in size in the anal canal and peripheral discontinuous melanin depositions. **c** Histopathological findings demonstrated diffuse proliferation of atypical cells with prominent nuclei of varying size accompanied by melanin depositions. **d** Positive for HMB-45. **e** Positive for S-100. **f** Positive for SOX-10

A 51-year-old female patient with a history of bipolar disorder and hyperlipidemia underwent a colonoscopy after a fecal occult blood test returned positive. Colonoscopy revealed a 30-mm-sized semi-pedunculated tumor in the lower rectum (Rb) and a black protruding lesion extending upwards into the anal canal. Based on these findings, ARMM was diagnosed (Fig. 1a, b). A laparoscopic rectal amputation was performed, and pathological analysis of tissue specimens revealed peripheral discontinuous melanin deposition (Fig. 2a, b). The tumor had invaded the submucosa, and one lymph node metastasis was detected. Histopathological findings indicated diffuse proliferation of atypical cells with prominent nuclei of varying size accompanied by melanin deposition (Fig. 2c). Immunostaining was positive for HMB-45 (Fig. 2d), S-100 (Fig. 2e), and SOX-10 (Fig. 2f). Based on these findings, stage III ARMM was diagnosed, and the patient was followed up without postoperative adjuvant chemotherapy.

**Fig. 3:**
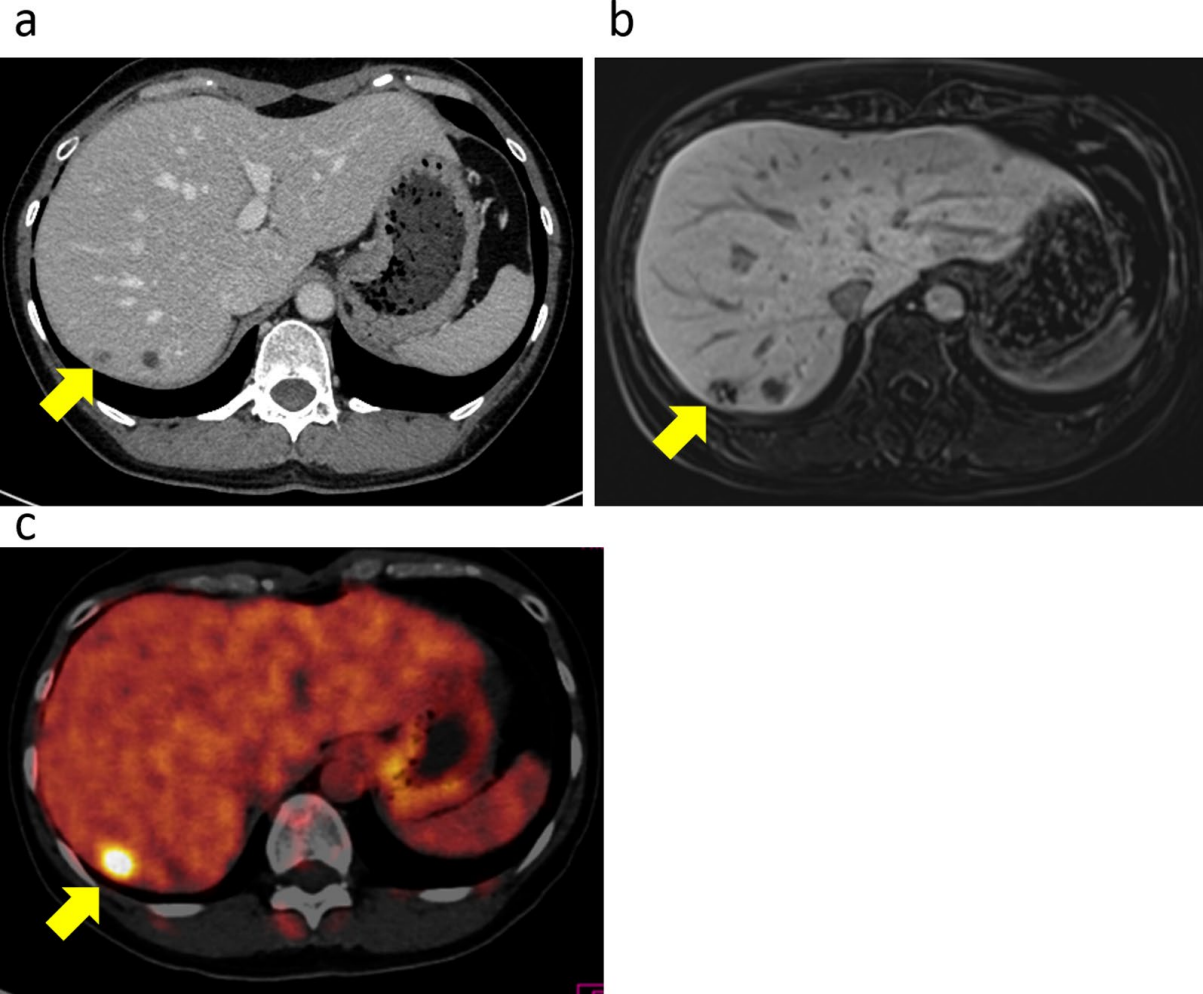
Imaging findings of the liver metastasis. **a** CT demonstrated a 14-mm-sized tumor with a faint contrast effect on the margin of liver S7. **b** The tumor showed a low signal intensity in the hepatocellular phase of Gd-EOB-DTPA-enhanced MRI. **c** The tumor showed strong accumulation of FDG on PET–CT

At postoperative months 3 and 8, CT revealed no recurrence. At postoperative month 11, CT revealed a 14-mm-diameter tumor with a faint contrast effect at the margin of S7 (Fig. 3a). The tumor showed low signal intensity in the hepatocellular phase of gadolinium-ethoxybenzyl-diethylenetriaminepentaacetic acid (Gd-EOB-DTPA)-enhanced magnetic resonance imaging (MRI) (Fig. 3b), and positron emission tomography (PET)–CT indicated a large accumulation of FDG (Fig. 3c). Based on these findings, a liver metastasis of ARMM was diagnosed.

Surgical resection is recommended for postoperative oligometastatic recurrences of malignant melanoma [5]. In the present patient, a single liver metastasis recurred 11 months after surgery for the primary lesion. A complete resection was possible via minimally invasive laparoscopic surgery; therefore, we decided to perform a laparoscopic partial resection of S7. The operative time was 4 h 8 min, and the amount of bleeding was 30 g. The postoperative course was uneventful, and the patient was discharged on postoperative day 7.

**Fig. 4:**
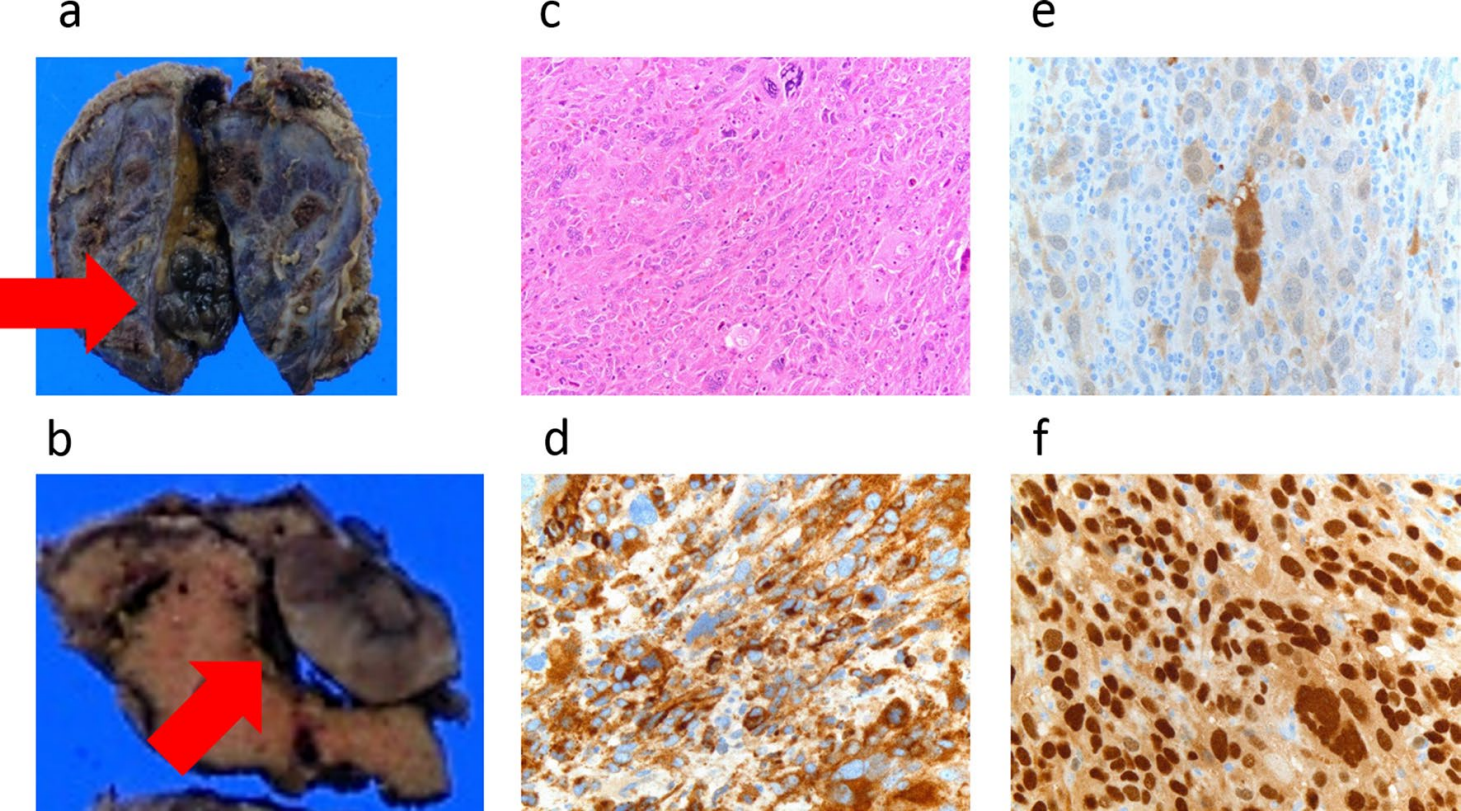
Macroscopic findings, histopathological findings, and immunostaining findings of the liver metastasis. **a**, **b** Pathological specimens revealed a 16-mm-diameter solid nodule accompanied by melanin depositions. **c** Histopathological findings demonstrated diffuse proliferation of atypical cells with prominent nuclei of varying size accompanied by melanin deposition. **d** Positive for HMB-45. **e** Positive for S-100. **f** Positive for SOX-10

Macroscopically, a 16-mm solid nodule was observed together with melanin deposition (Fig. 4a–c). Immunostaining was positive for HMB-45 (Fig. 4d), S-100 (Fig. 4e), and SOX-10 (Fig. 4f), confirming the diagnosis of a liver metastasis of ARMM. Nivolumab was started as adjuvant chemotherapy, and 7 months after the surgery for the metastasis, the patient is still alive without any recurrences.

## Discussion

Malignant melanoma is a mesenchymal tumor originating in melanocytes. It may occur in the skin, retina, head and neck area or gastrointestinal tract [4]. ARMM is a rare disease, accounting for 1% of malignant melanoma cases and 0.5–2% of anorectal malignancies [1]. The prognosis of ARMM is poor, with a 5-year survival rate of 25% and a median survival of 16–28 months [1, 2].

For malignant melanoma with distant metastases, the first-line treatment is chemotherapy, including targeted drugs and immune checkpoint inhibitors. When nivolumab, an anti-PD-1 antibody, was administered to patients with malignant melanoma with distant metastases, the 1-year survival rate was 72.9% [6]. When ipilimumab, an anticytotoxic T-lymphocyte antigen-4 (anti-CTLA-4) monoclonal antibody, was administered at 10 mg/kg to a similar group of patients, the median overall survival was 15.7 months and the 5-year survival rate was 25% [7].

On the other hand, surgical resection may be considered for a distant oligometastatic recurrence of malignant melanoma if total resection of the lesion is possible [5, 8–11]. In such cases, the 1-year survival rate is reportedly 51%, and the 2-year survival rate is 38% [5]. Patients in past reports who underwent a complete resection of liver metastases of malignant melanoma had a median survival time of 27.7 months and a 5-year survival rate of 33% [8].

Although some studies have reported resection of liver metastases of malignant melanoma [12, 13], none so far have reported resection of a liver metastasis of ARMM.

In the present instance, surgery was chosen as the treatment modality because the lesion was a single oligometastatic recurrence of liver metastasis occurring 11 months after resection of the primary ARMM lesion. The procedure was able to be performed via laparoscopic surgery, which is less invasive than other surgical techniques.

Nivolumab has been shown to be effective as postoperative adjuvant therapy in patients with melanoma with completely resected lymph node metastases or distant metastases. In a previous study where nivolumab was given as adjuvant chemotherapy for one year after complete resection of stage III and IV malignant melanoma, the recurrence-free survival rate was 51.7% and the overall survival rate was 77.9% after 4 years [14].

In recent years, various anticancer agents and molecular-targeted agents have appeared in the adjuvant chemotherapy arsenal against malignant melanoma, including dabrafenib plus trametinib [15, 16] and pembrolizumab [17]. In the treatment of ARMM, multidisciplinary treatment combining surgery and chemotherapy for metastatic lesions has the potential to improve the prognosis.

## Conclusions

We reported a case of ARMM in which the primary lesion was resected laparoscopically. A liver metastasis was also subsequently resected laparoscopically. This is the first report of resection of liver metastases of ARMM. Although the prognosis of ARMM is poor, surgery should be considered in cases of oligometastasis.

## Data Availability

Data sharing is applicable to this article.
