# Correction: A case of laparoscopic partial hepatic S7 resection for postoperative liver metastasis of rectal malignant melanoma

**DOI:** 10.1186/s40792-024-02042-1

**Published:** 2024-10-29

**Authors:** Makoto Takahashi, Yasuhiro Morita, Tatsuya Hayashi, Susumu Yanagibashi, Shunsuke Sato, Shu Sasaki, Kunio Takuma, Haruka Okada

**Affiliations:** 1 Department of Surgery, Tokyo Metropolitan Tama Medical Center, 2-8-29 Musashidai, Fuchu, Tokyo, 183-8524 Japan; 2 Department of Pathology, Tokyo Metropolitan Tama Medical Center, Tokyo, Japan

**Correction: Surg Case Rep (2021) 7:230** 10.1186/s40792-021-01316-2

Following publication of the original article [1], the authors reported a typo in the 4th author’s name.

The originally published name was: Susumu Yanagibasi

The correct name should be: Susumu Yanagibashi

The original article [1] has been updated.
